# Editorial: Mechanisms and New Targets for the Treatment of Chronic Pain

**DOI:** 10.3389/fphar.2020.600037

**Published:** 2020-09-29

**Authors:** Francisco Rafael Nieto, Sonja Maksim Vuckovic, Milica S. Prostran

**Affiliations:** ^1^ Department of Pharmacology, School of Medicine, University of Granada, Granada, Spain; ^2^ Institute of Neuroscience, Biomedical Research Center, University of Granada, Granada, Spain; ^3^ Biosanitary Research Institute, University Hospital Complex of Granada, Granada, Spain; ^4^ Department of Pharmacology, Clinical Pharmacology and Toxicology, Faculty of Medicine, University of Belgrade, Belgrade, Serbia

**Keywords:** chronic pain, persistent pain, neuropathic pain, musculoskeletal pain, visceral pain, cancer-related pain, analgesics, pain

## Background

Nociceptive pain has a physiological protective role in preventing tissue injury. However, pain can become chronic due to a multitude of pathophysiological states, such as: inflammation, nerve injury, tumors, infections, autoimmune diseases, and vascular and metabolic disorders. These pathological states can trigger alterations of the pain pathways that can lead to painful hypersensitivity, and in such circumstances, pain loses its protective role and instead, becomes chronic, pathologic, and it can be extremely debilitating for people who suffer from it (Basbaum et al., 2009). Chronic pain has recently been defined as pain that persists or recurs for more than 3 months (Treede et al., 2019)

Chronic pain is a very significant health problem around the world. Although its prevalence is difficult to calculate, it has been estimated that a 10% of adults are diagnosed with chronic pain each year (Goldberg and McGee, 2011), and in a very recently meta-analysis, the prevalence of chronic pain in the general population of developing countries was estimated at 18% (Sá et al., 2019). However, the management of chronic pain is still no fully satisfactory probably due to the variety of persistent pain conditions with different etiologies, such as musculoskeletal (McWilliams and Walsh, 2017), neuropathic (Alles and Smith, 2018), visceral (Gebhart and Bielefeldt, 2016) and cancer-related (Lam, 2016) pain, whose pathophysiological mechanisms are only partially known. In addition, current analgesics for pathologic pain are not totally effective in all patients and are associated with serious side effects and/or abuse and dependence problems, in fact, opioids still keep an important role in the treatment of certain chronic pain conditions (Finnerup et al., 2015; Noori et al., 2019). Non-pharmacological treatments for chronic pain relief, such as acupuncture and different neurostimulation techniques might have some therapeutic benefits, although the clinical efficacy of these complementary therapeutic approaches are difficult to prove (Coutaux, 2017; Lefaucheur, 2019). Therefore, there is a huge need for new effective therapies with less adverse effects for the control and prevention of the different types of chronic pain.

Chronic pain can emerge as a consequence of dysfunction of the nociceptive circuits at any level of the nervous system, resulting in an increased perception of pain (hyperalgesia and allodynia), and even spontaneous pain. Tissue damage and inflammation lead to a reduced threshold and increased sensitivity of the peripheral terminals of the nociceptors, resulting in pain hypersensitivity – a process known as peripheral sensitization (Costigan et al., 2009). On the other hand, central sensitization represents a phenomenon of enhanced function of neurons and circuits of central pain pathways, caused by increased membrane excitability and synaptic efficacy and by reduced inhibition (Latremoliere and Woolf, 2009). Peripheral and central sensitization is a manifestation of the extraordinary plasticity of the somatosensory nervous system in response to inflammation or injury and they are major contributor of chronic pain (Woolf and Salter, 2000). Neuronal functional changes, including alterations in neurotransmitter synthesis and signaling, and changes in expression and trafficking of receptors and ion channels can trigger the sensitization of the pain pathways (Costigan et al., 2009; Latremoliere and Woolf, 2009). In addition, accumulating evidence suggests that non-neuronal cells mainly immune and glial cells play active roles in the pathogenesis and resolution of chronic pain by releasing neuroactive mediators that modulate pain (Hore and Denk, 2019). Among these non-neuronal cells, satellite glial cells, macrophages and T cells in the peripheral nervous system, and microglia and astrocytes in the central nervous system, are the most studied by far. Interestingly, nociceptive neurons can also release active substances on glial and immune cells, which can also contribute to the neuroinflammatory process that is involved in chronic pain (Ji et al., 2016). Therefore, there is an authentic cross-talk between neurons and immune and glial cells which has attracted the attention of numerous pain scientists in the last years to increase our knowledge of how neuroimmune and neuroglial mechanisms contribute to chronic pain states.

## Overview of the Articles Included in This Research Topic

This Research Topic includes twenty four articles that address different aspect of the pathophysiology and treatment of chronic pain. Most of the studies are basic research with experimental animals, but our Research Topic also includes clinical research. In addition, the reader will also find several reviews and meta-analysis addressing very interesting subjects.

Among the basic research studies, three articles focused on neuropathic pain induced by chemotherapy, one of the most prevalent adverse effects of cancer patients treated with antineoplastic drugs (Sisignano et al., 2014). Kim et al. showed that a selective phosphodiesterase (PDE)-4 inhibitor called Rolipram attenuated mechanical hypersensitivity induced by the antitumour drug paclitaxel in rats. The mechanism involved seems to be related to the decreased on the expression of inflammatory cytokines in the DRG induced by paclitaxel. In another article conducted by Meng et al., it was shown that the antidepressant drug duloxetine (a serotonin-norepinephrine reuptake Inhibitor) was effective in reducing neuropathic pain induced by two antineoplastic drugs (paclitaxel or oxaliplatin) in mice. Duloxetine is considered a first line drug for the treatment of chemotherapy-induced painful neuropathies, although the scientific evidence to support its efficacy in these neuropathies is still limited (Ibrahim and Ehrlich, 2020). Meng et al. provided important evidence on the mechanisms involved in the ameliorative effects of duloxetine in chemotherapy-induced neuropathic pain that point to an inhibition of the activation of p38 MAPK and NF-kB induced by chemotherapy, thus reducing the inflammatory response in the DRG. In addition, this study showed that duloxetine did not affect the antitumor activity of oxaliplatin and paclitaxel, which is a key issue for the pharmacological management of chemotherapy-induced peripheral neuropathies. Starobova et al. established a new model of peripheral neuropathy induced by vincristine based on the local administration of this drug into the hind paw of mice. They compared the resulting pain phenotypes from this new model to that of a conventional model based on systemic administration of vincristine. They found that mechanical allodynia was decreased in mice lacking Toll-like receptor 4, as well as in mice treated with the antibiotic minocycline, which has been shown to modulate the activation of microglia and immune cells.

Two studies evaluated the analgesic effects of natural compounds on models of neuropathic pain induced by chronic constriction injury (CCI) of the sciatic nerve. Jin et al. demonstrated that systemic administration of koumine (an indole alkaloid presents in *Gelsemium elegans*) reduced CCI-induced mechanical allodynia in rats by reducing astrocyte-mediated neuroinflammation. In the study conducted by Fotio et al. was shown that a combination of several natural products, including N-Palmitoylethanolamide (PEA), beta-caryophyllene, carnosic acid and myrrh extract administered orally, was as effective as gabapentin, a first line drug for treating neuropathic pain (Finnerup et al., 2015), reducing pain behaviors induced by CCI in mice.

Two other articles evaluate the effects of natural plant-derived products on different pain models. Uddin et al. described the antinociceptive effects of the methanol extract of *Anisomeles indica* on two classical model of chemical-induced pain such as, acetic acid-induced writhing test and formalin-induced licking test. Huang et al. found that Bulleyaconitine A, a diterpenoid alkaloid isolated from Aconitum Bulleyanum, produced antinociceptive effect in the acetic acid-induced writhing test in rats. In addition, this compound showed anxiolytic effects and improved gastrointestinal function in rats with chronic visceral pain.

Other basic research studies collected in this Research Topic evaluated the analgesic effects of several drugs on different pain models. Thus, Zhang et al. described that duloxetine had analgesic effects on formalin-induced pain hypersensitivity through increasing the concentration of serotonin in the central nucleus of amygdala (CeA). Hu et al. described that the local administration of TRPV1 antagonist had analgesic effects on pain hypersensitivity associated to a rat model of complex regional pain syndrome (CRPS) induced by ischemia. This study suggests that TRPV1 channel might have an important role on this pain syndrome, whose pathophysiology remain poorly understood (Eldufani et al., 2020). Idris et al. showed that two synthetic benzimidazole derivatives attenuated morphine-induced paradoxical pain in mice. Srebro et al. showed that systemic administration of tramadol had both prophylactic and therapeutic analgesic effects in inflammatory pain and edema induced by intraplantar injection of carrageenan. Interestingly, they found that the co-administration of a fixed dose of tramadol with different doses of magnesium sulfate led to a dose-dependent enhancement of the analgesic effect of tramadol, opening the door to the use of magnesium as adjuvant therapy along with tramadol to counteract inflammatory pain. Magnesium ion acts as a blocker of the N-methyl-D-aspartate (NMDA) receptor in the central nervous system and it is well known that NMDA receptor activation is key in the central sensitization process (Latremoliere and Woolf, 2009).

Another study conducted by Metcalf et al. used a non-pharmacological approach to demonstrate that music can enhance analgesia and antiseizure effects of several drugs in animal models of pain and epilepsy. The playlist used in the experiments comprised several Mozart`s compositions. Although the analgesic effects of music “per se” (Lunde et al., 2019) or as adjunct to analgesic drugs (Chai et al., 2017) have been previously described in patients, Metcalf et al. presented the first animal study on music-enhanced antinociceptive activity of analgesic drugs.

This Research Topic also includes a technology report performed by González-Cano et al. which described a software to simplify the process of calculating 50% mechanical threshold values, when using calibrated von Frey filaments with the up–down testing paradigm. This article can be of interest for scientists working on basic pain research with rodents, as von Frey filaments are by far the tool used most frequently to assess tactile allodynia in mice and rats (González-Cano et al., 2020). They present an open-source computer program that can read handwritten von Frey result sheets and translate these measurements into mechanical hypersensitivity threshold values, eliminating the chance of a manual calculation error.

There are also several clinical reports in this Research Topic. Thibaut et al. presented a pilot crossover randomized controlled study to evaluate the effects of transcranial direct current stimulation and transcranial pulsed current stimulation on brain oscillations in patients with chronic visceral pain. Knezevic et al. conducted an open-labeled pilot study to determine the efficacy and safety of a new formulation of gabapentin-extended release in patients with postamputation pain. Gabapentin is a first-line drug for the treatment of neuropathic pain (Finnerup et al., 2015) and Knezevic et al. demonstrated for the first time, that once-daily dosing of gabapentin-extended release shows significant improvement in pain severity and functional status in these patients, with rates of side effects similar to previously published literature. Shan et al. assessed the current available evidence of a traditional Chinese medicine herb from the rhizome of *Ligusticum chuanxiong*, for the treatment of migraine. The results of this systematic review article indicated that this herb can reduce frequency, duration and pain severity of migraine, with a low rate of side effects. Another systematic review article conducted by García-Henares et al. showed that low doses of intravenous ketamine (a NMDA receptor antagonistin) reduced morphine consumption and pain intensity scores after remifentanil-based general anesthesia for major and minor surgery in adults. This report also showed that ketamine did not increase the incidence of adverse effects.

Finally, the present Research Topic also offers to the reader a good number of review articles that provide an update on the pathophysiology and treatment of different type of chronic pain. Valente performed a mini review about the pharmacology of pain associated with the monoiodoacetate (MIA) model of osteoarthritis. This model consists of intra-articular injection of MIA to rodents and results in histopathological changes and functional disability that mimic to some extend some of the features of patients with osteoarthritis. Vučković et al. conducted an extensive narrative review about the role of cannabinoids in the treatment of pain. This article includes preclinical and clinical research about the pharmacodynamics, pharmacokinetics, efficacy, safety and tolerability of these compounds. Joksimovic et al. addressed the role of neurosteroids in the management of pain with an interesting review that focuses on their involvement in the pain pathway, their potential use as analgesics in different models of pain, as well as the future therapeutic perspectives. Llorián-Salvador and González-Rodríguez carried out an opinion article about the involvement of the vascular endothelial growth factor (VEGF) in several types of pain. VEGF is an important mediator with pro-angiogenic properties, being an important regulatory factor in tumor angiogenesis what explains the use of anti-VEGF therapies for the treatment of cancer. However, VEGF has been proposed as a potential key factor in the establishment and maintenance of chronic pain.

There are also three review articles with a more clinical perspective. Vigano et al. performed an update on the role of neurophysiological abnormalities and maladaptive plasticity on the treatment of migraine with neuromodulation. They reviewed the available evidence from therapeutic and physiological studies using neuromodulation in chronic migraine. Stamenkovic et al. carried out a narrative review to analyze preventive measures to avoid the development of chronic pain in intensive care unit (ICU) patients. In addition, they outlined possible management options to minimize both chronic pain and long term opioid use following ICU discharge. Knezevic et al. performed a review about the role of corticosteroids in the treatment of chronic pain. These drugs have been widely used in the treatment of back pain and osteoarthritis over the past decades. However, their use has been also questioned due to the serious adverse events of corticosteroids, and the emergence of new therapeutic options.

## Author Contributions

All authors contributed to the article and approved the submitted version.

## Conflict of Interest

The authors declare that the research was conducted in the absence of any commercial or financial relationships that could be construed as a potential conflict of interest.

## References

[B1] Alles S. R. A. Smith P. A. (2018). Etiology and Pharmacology of Neuropathic Pain. Pharmacol. Rev. 70, 315–347. 10.1124/pr.117.014399 29500312

[B2] Basbaum A. I. Bautista D. M. Scherrer G. Julius D. (2009). Cellular and molecular mechanisms of pain. Cell 139, 267–284. 10.1016/j.cell.2009.09.028 19837031 PMC2852643

[B3] Chai P. R. Carreiro S. Ranney M. L. Karanam K. Ahtisaari M. Edwards R. (2017). Music as an Adjunct to Opioid-Based Analgesia. J. Med. Toxicol. 13, 249–254. 10.1007/s13181-017-0621-9 28646359 PMC5570730

[B4] Costigan M. Scholz J. Woolf C. J. (2009). Neuropathic pain: a maladaptive response of the nervous system to damage. Annu. Rev. Neurosci. 32, 1–32. 10.1146/annurev.neuro.051508.135531 19400724 PMC2768555

[B5] Coutaux A. (2017). Non-pharmacological treatments for pain relief: TENS and acupuncture. Joint Bone Spine 84, 657–661. 10.1016/j.jbspin.2017.02.005 28219657

[B6] Eldufani J. Elahmer N. Blaise G. (2020). A medical mystery of complex regional pain syndrome. Heliyon 6:e03329. 10.1016/j.heliyon.2020.e03329 32149194 PMC7033333

[B7] Finnerup N. B. Attal N. Haroutounian S. McNicol E. Baron R. Dworkin R. H. (2015). Pharmacotherapy for neuropathic pain in adults: a systematic review and meta-analysis. Lancet Neurol. 14, 162–173. 10.1016/S1474-4422(14)70251-0 25575710 PMC4493167

[B8] Gebhart G. F. Bielefeldt K. (2016). Physiology of Visceral Pain. Compr. Physiol. 6, 1609–1633. 10.1002/cphy.c150049 27783853

[B9] Goldberg D. S. McGee S. J. (2011). Pain as a global public health priority. BMC Public Health 11:770. 10.1186/1471-2458-11-770 21978149 PMC3201926

[B10] González-Cano R. Montilla-García Á Ruiz-Cantero M. C. Bravo-Caparrós I. Tejada MÁ Nieto F. R. (2020). The search for translational pain outcomes to refine analgesic development: Where did we come from and where are we going? Neurosci. Biobehav. Rev. 113, 238–261. 10.1016/j.neubiorev.2020.03.004 32147529

[B11] Hore Z. Denk F. (2019). Neuroimmune interactions in chronic pain - An interdisciplinary perspective. Brain Behav. Immun. 79, 56–62. 10.1016/j.bbi.2019.04.033 31029795

[B12] Ibrahim E. Y. Ehrlich B. E. (2020). Prevention of Chemotherapy-Induced Peripheral Neuropathy: A Review of Recent Findings. Crit. Rev. Oncol. Hematol. 145:102831. 10.1016/j.critrevonc.2019.102831 31783290 PMC6982645

[B13] Ji R. R. Chamessian A. Zhang Y. Q. (2016). Pain regulation by non-neuronal cells and inflammation. Science 354, 572–577. 10.1126/science.aaf8924 27811267 PMC5488328

[B14] Lam D. K. (2016). Emerging Factors in the Progression of Cancer-Related Pain. Pain Manag. 6, 487–496. 10.2217/pmt-2015-0003 27150228

[B15] Latremoliere A. Woolf C. J. (2009). Central sensitization: a generator of pain hypersensitivity by central neural plasticity. J. Pain. 10 (9), 895–926. 10.1016/j.jpain.2009.06.012 19712899 PMC2750819

[B16] Lefaucheur J. P. (2019). Transcranial magnetic stimulation. Handb. Clin. Neurol. 160, 559–580. 10.1016/B978-0-444-64032-1.00037-0 31277876

[B17] Lunde S. J. Vuust P. Garza-Villarreal E. A. Vase L. (2019). Music-induced analgesia: how does music relieve pain? Pain 160, 989–993. 10.1097/j.pain.0000000000001452 30507782

[B18] McWilliams D. F. Walsh D. A. (2017). Pain mechanisms in rheumatoid arthritis. Clin. Exp. Rheumatol. 35 Suppl 107, 94–101. 28967354

[B19] Noori S. A. Aiyer R. Yu J. White R. S. Mehta N. Gulati A. (2019). Nonopioid versus opioid agents for chronic neuropathic pain, rheumatoid arthritis pain, cancer pain and low back pain. Pain Manag. 9, 205–216. 10.2217/pmt-2018-0052 30681031

[B20] Sá K. N. Moreira L. Baptista A. F. Yeng L. T. Teixeira M. J. Galhardoni R. (2019). Prevalence of chronic pain in developing countries: systematic review and meta-analysis. Pain Rep. 4, e779. 10.1097/PR9.0000000000000779 31984290 PMC6903356

[B21] Sisignano M. Baron R. Scholich K. Geisslinger G. (2014). Mechanism-based treatment for chemotherapy-induced peripheral neuropathic pain. Nat. Rev. Neurol. 10, 694–707. 10.1038/nrneurol.2014.211 25366108

[B22] Treede R. D. Rief W. Barke A. Aziz Q. Bennett M. I. Benoliel R. (2019). Chronic pain as a symptom or a disease: the IASP Classification of Chronic Pain for the International Classification of Diseases (ICD-11). Pain 160, 19–27. 10.1097/j.pain.0000000000001384 30586067

[B23] Woolf C. J. Salter M. W. (2000). Neuronal plasticity: increasing the gain in pain. Science 288, 1765–1769. 10.1126/science.288.5472.1765 10846153

